# Association of low vitamin B12 status with incident dementia and stroke: an EHR database study

**DOI:** 10.3389/fnut.2026.1877529

**Published:** 2026-07-07

**Authors:** I-Wen Chen, Li-Chen Chang, Yi-Chen Lai, Kuo-Chuan Hung

**Affiliations:** 1 Department of Anesthesiology, Chi Mei Hospital, Liouying, Tainan, Taiwan; 2 Department of Anesthesiology, E-Da Hospital, I-Shou University, Kaohsiung, Taiwan; 3 Department of Anesthesiology, Chi Mei Medical Center, Tainan, Taiwan

**Keywords:** dementia, mild cognitive impairment, propensity score matching, stroke, vitamin B12, vitamin B12 deficiency

## Abstract

**Background:**

Epidemiological evidence linking low vitamin B12 status to dementia risk remains inconsistent, partly reflecting differences in exposure definitions, study designs, and analytical approaches. This study evaluated the association between low vitamin B12 status and long-term risks of incident dementia, related neurocognitive, and cerebrovascular outcomes.

**Methods:**

This propensity score–matched retrospective cohort study used de-identified electronic health record data from the TriNetX Global Collaborative Network. Adults aged ≥50 years with two vitamin B12 measurements <300 pg./mL within a 2-year window were matched 1:1 with patients who had two vitamin B12 measurements of 300–900 pg./mL using the same framework. A 1-year landmark period was applied, with outcome follow-up beginning on day 366 after the index date. The primary outcome was incident all-cause dementia over 10 years. Secondary outcomes included Alzheimer’s disease, vascular dementia, other dementia subtypes, mild cognitive impairment, stroke, and all-cause mortality. An exposure-gradient analysis was performed among patients with vitamin B12 deficiency, defined as two measurements <200 pg./mL.

**Results:**

After matching, 129,159 patients remained in each group. Low vitamin B12 status was associated with a higher risk of all-cause dementia (Hazard ratio [HR] 1.33, 95% confidence interval [CI] 1.27–1.39, *p* < 0.001). Associations were also observed for Alzheimer’s disease, vascular dementia, and other dementia subtypes (HRs 1.31–1.34), mild cognitive impairment (HR 1.33), stroke (HR 1.31), and all-cause mortality (HR 1.23; all *p* < 0.001). Among patients with vitamin B12 deficiency, the association with all-cause dementia was stronger (HR 1.64, *p* < 0.001). Results were consistent across six prespecified sensitivity analyses and sex-stratified subgroup analyses.

**Conclusion:**

Low vitamin B12 status was associated with an increased long-term risk of incident all-cause dementia, with a numerically stronger association among patients with vitamin B12 deficiency. Given the observational design and potential residual bias, these findings should be interpreted as hypothesis-generating rather than causal. Further prospective studies with serial biomarker monitoring and interventional studies of vitamin B12 correction are needed to clarify this association.

## 1 Introduction

Dementia affects over 55 million people worldwide and remains a leading cause of disability among older adults (1, 2). Because disease-modifying therapies remain limited, increasing attention has focused on modifiable risk factors that may be detectable before irreversible neurodegeneration occurs (3). Nutritional deficiencies are particularly relevant because they are common in older adults, measurable in routine clinical practice, and potentially reversible (4–6). Vitamin B12 is an essential cofactor for methionine synthase and methylmalonyl-CoA mutase, enzymes involved in myelin maintenance, one-carbon metabolism, and neurotransmitter synthesis (7–10). Sustained low vitamin B12 status may lead to accumulation of homocysteine and methylmalonic acid, which have been linked to neurotoxicity, impaired methylation, and cerebrovascular endothelial dysfunction (11–14). These mechanisms provide biological plausibility for associations between low vitamin B12 status, dementia, and cerebrovascular outcomes such as stroke.

Despite this biological rationale, epidemiological evidence linking low vitamin B12 status to dementia risk remains inconsistent. Meta-analyses have suggested that elevated homocysteine (a functional marker related to B-vitamin status) is associated with higher dementia risk (15), whereas serum vitamin B12 concentrations alone have not shown a consistent association with cognitive decline or dementia (16, 17). A large registry-based propensity score analysis including more than 53,000 patients with low plasma vitamin B12 levels and up to 15 years of follow-up found no association between low plasma vitamin B12 status and dementia risk (18). This discrepancy may reflect two specific methodological issues. First, a single vitamin B12 measurement may misclassify transient laboratory variation as chronic low vitamin B12 status, whereas persistent deficiency may be more biologically relevant to neurocognitive risk. Second, the absence of a lag or landmark period may allow prodromal cognitive decline, frailty, or reduced nutritional intake to influence both vitamin B12 testing and subsequent dementia diagnosis (19, 20), thereby obscuring temporal interpretation. Therefore, a longitudinal design incorporating repeated vitamin B12 measurements and a predefined landmark period is needed to clarify whether sustained low vitamin B12 status is associated with subsequent dementia risk.

Addressing these knowledge gaps requires a study design that better distinguishes sustained low vitamin B12 status from transient laboratory variation and establishes clearer temporal separation between vitamin B12 assessment and subsequent outcome ascertainment. This study therefore aimed to evaluate the association of persistent low vitamin B12 status with long-term risks of incident dementia and related cerebrovascular outcomes among adults aged 50 years or older.

## 2 Methods

### 2.1 Data source and ethical statement

This propensity score–matched retrospective cohort study was conducted using de-identified electronic health record data from the TriNetX Global Collaborative Network, a federated research platform comprising 173 healthcare organizations. The TriNetX database has been widely used in epidemiological and real-world evidence studies across multiple clinical fields (21–23). TriNetX provides investigators with access only to aggregated, de-identified patient-level data, and no direct patient identifiers are available for review or extraction. Given the use of de-identified data, the Institutional Review Board of Chi Mei Medical Center waived the requirement for individual informed consent. All study procedures were performed in accordance with the Declaration of Helsinki and relevant institutional regulations governing retrospective observational research.

### 2.2 Exposure definition

Eligible patients were adults aged 50 years or older with serum, plasma, or blood vitamin B12 measurements recorded between January 1, 2010, and December 31, 2023. Patients were assigned to the low vitamin B12 cohort if they had two vitamin B12 measurements <300 pg./mL within a 2-year window. In this study, vitamin B12 levels <300 pg./mL were defined as low vitamin B12 status, whereas levels <200 pg./mL were defined as vitamin B12 deficiency (24). The index date was defined as the date of the second qualifying measurement, thereby reflecting persistent low vitamin B12 status rather than a transient laboratory fluctuation. The reference cohort comprised patients whose vitamin B12 levels were 300–900 pg./mL using the same two-measurement framework. For the reference cohort, the index date was likewise defined as the date of the second qualifying normal vitamin B12 measurement, ensuring comparable temporal alignment of exposure classification, propensity score matching, the landmark period, and outcome follow-up between cohorts.

### 2.3 Exclusion criteria

To reduce misclassification related to acute illness, patients were excluded if acute kidney failure, sepsis, severe sepsis, or critical care services were documented within 1 month before a qualifying vitamin B12 measurement. Additional exclusions included conditions that could independently cause cognitive decline or confound the exposure–outcome relationship, such as a prior diagnosis of all-cause dementia (Alzheimer’s disease, vascular dementia, dementia in other diseases, or unspecified dementia), other degenerative diseases of the nervous system, prior cerebrovascular events (cerebral infarction, intracerebral hemorrhage, or transient ischemic attack), advanced chronic kidney disease, end-stage renal disease, dialysis dependence, Parkinson’s disease, bipolar disorder, schizophrenia or related psychotic disorders, prior or current bariatric surgery status, intracranial injury, and central nervous system neoplasms (benign, malignant, uncertain, or unspecified).

### 2.4 Landmark analysis

A 1-year landmark design was used to strengthen temporal alignment between vitamin B12 status and subsequent dementia risk. Eligible patients were required to survive and remain free of dementia for 365 days after the index date; outcome follow-up was then initiated on day 366. This approach was intended to reduce reverse causation, particularly the possibility that prodromal cognitive symptoms or declining general health prompted vitamin B12 testing before a dementia diagnosis. By excluding dementia events occurring within the first year, the analysis also minimized the influence of early diagnoses more likely attributable to pre-existing neurocognitive decline or baseline disease severity rather than vitamin B12 status itself. In addition, requiring survival through the landmark period helped preserve the comparability achieved by propensity score matching at the index date by limiting bias from differential early mortality between groups.

### 2.5 Data collection and propensity score matching

To reduce baseline differences between the low vitamin B12 and reference cohorts, we applied 1:1 propensity score matching using a greedy nearest-neighbor approach with a caliper of 0.1 pooled standard deviations. Matching was performed without replacement. Propensity scores were estimated using baseline covariates assessed before the index date, and covariates were entered as main effects without interaction terms. No propensity score truncation was applied. Laboratory variables were analyzed using available data without imputation, consistent with the data structure of TriNetX. Time-dependent covariates were not modeled. Propensity scores were estimated using a prespecified set of clinically relevant covariates that were considered potential confounders because of their associations with vitamin B12 status, dementia risk, or both. These variables included demographic characteristics, cardiometabolic and vascular diseases, neuropsychiatric disorders, medication exposures, and laboratory measures capturing renal function, systemic inflammation, nutritional status, glycemic profile, hematologic status, and body composition (Supplementary Table 1). After matching, baseline balance between cohorts was evaluated using standardized mean differences; an absolute standardized mean difference of <0.10 was considered to indicate adequate balance. The overlap and redistribution of propensity scores before and after matching were further examined using density plots.

### 2.6 Primary and secondary outcomes

The primary endpoint was newly diagnosed all-cause dementia after the landmark period. Dementia was identified using ICD-10-CM codes for Alzheimer’s disease (G30), vascular dementia (F01), dementia in other diseases classified elsewhere (F02), and unspecified dementia (F03). To ensure temporal separation between vitamin B12 status and outcome occurrence, dementia ascertainment started only after the 1-year landmark period and continued for a maximum of 10 years.

Secondary endpoints were included to provide a broader assessment of neurocognitive, cerebrovascular, and survival outcomes. These comprised specific dementia categories, including Alzheimer’s disease, vascular dementia, and other or unspecified dementia; mild cognitive impairment (G31.84); stroke; and all-cause mortality. Patients were followed until the first diagnosis of the outcome being evaluated, death, the last recorded encounter in the electronic health record, or the end of the 10-year follow-up period, whichever occurred first.

### 2.7 Positive and negative control outcomes

To examine the credibility and specificity of the study findings, we also performed prespecified control outcome analyses. Vitamin B12 deficiency anemia was chosen as the positive control outcome because it represents a recognized clinical manifestation of prolonged vitamin B12 deficiency. Acute appendicitis (ICD-10-CM: K35) was chosen as the negative control outcome because it is not expected to be biologically related to vitamin B12 status. Together, these control outcomes were used to assess whether the observed associations were aligned with expected biological relationships rather than reflecting nonspecific bias or differential outcome ascertainment.

### 2.8 Sensitivity analyses, subgroup analyses, and multivariable regression

Robustness was evaluated through six prespecified sensitivity analyses: Model I excluded patients who died during follow-up; Model II was restricted to patients with more than one healthcare visit during follow-up; Model III excluded patients who developed vitamin B12 deficiency anemia during follow-up; Model IV excluded patients with a prior serum albumin below 3.5 g/dL; Model V excluded patients with a prior history of chronic kidney disease; and Model VI excluded patients with a prior history of neoplasm.

Sex-specific subgroup analyses were performed by repeating propensity score matching and outcome analyses independently within each stratum. Effect modification by sex was examined using interaction tests. In addition to the propensity score–matched analysis, we constructed sequential multivariable Cox proportional hazards models to examine the robustness of the association between low vitamin B12 status and incident all-cause dementia. Covariates were introduced in a stepwise manner across models, allowing assessment of whether the observed association persisted after progressive adjustment for demographic characteristics and clinical comorbidities potentially related to both vitamin B12 status and dementia risk.

### 2.9 Exposure-gradient analysis

To explore a potential exposure-gradient relationship, the risk of all-cause dementia was assessed separately in patients with vitamin B12 deficiency, defined as two measurements below 200 pg./mL within a 2-year window. The exposure classification, exclusion criteria, landmark period, propensity score matching procedures, and outcome definitions were identical to those of the main analysis. The magnitude of the association in this deficiency subgroup was compared with that of the primary analysis to evaluate whether a gradient relationship was present.

### 2.10 Statistical analysis

Time-to-event analyses were conducted using cause-specific Cox proportional hazards models, with results expressed as hazard ratios (HR) and 95% confidence intervals (CI). A cause-specific framework was chosen because the primary objective was to examine the etiological association between vitamin B12 status and subsequent dementia rather than to predict cumulative incidence in the presence of the competing risk of death. In this framework, deaths before dementia were treated as competing events that precluded subsequent dementia ascertainment and were censored at the time of death. Therefore, the estimated HRs should be interpreted as cause-specific associations among individuals who remained at risk, not as direct estimates of absolute cumulative incidence. The proportional hazards assumption was assessed using Schoenfeld residuals. Dementia-free survival was depicted using Kaplan–Meier curves, and between-group differences were evaluated with log-rank tests. E-values were calculated for the primary endpoint to quantify the minimum strength of association that an unmeasured confounder would need with both low vitamin B12 status and dementia to fully explain the observed result (25, 26). Statistical significance was set at a two-sided *α* of 0.05 for the primary analysis. Secondary, control, sensitivity, and subgroup analyses were considered exploratory, and no multiplicity adjustments were applied. All analyses used available data without imputation.

## 3 Results

### 3.1 Patient selection and baseline characteristics

**Figure 1:**
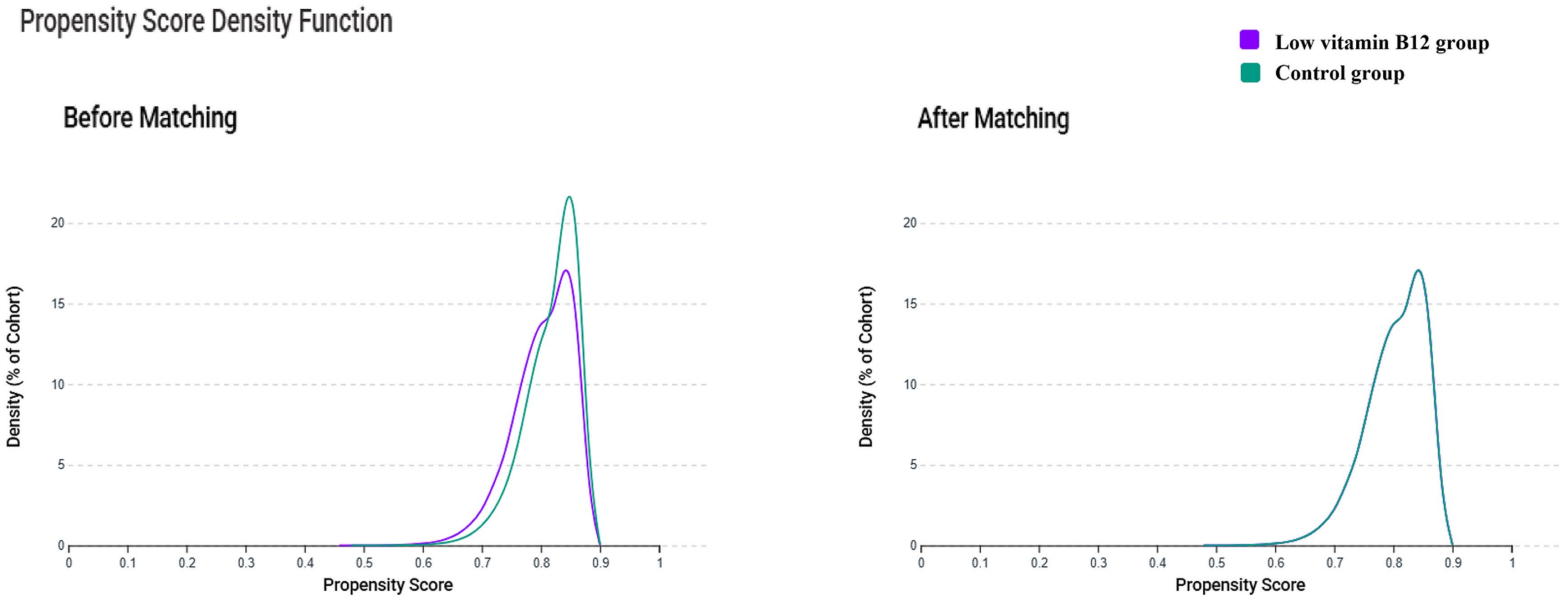
Propensity score density distributions before and after matching. Propensity score density distributions before and after matching. The figure demonstrates improved overlap between the low vitamin B12 and reference cohorts after 1:1 propensity score matching.

A total of 129,162 patients met the criteria for low vitamin B12 status, and 612,405 patients constituted the initial reference cohort with normal vitamin B12 levels. After 1:1 propensity score matching, 129,159 patients remained in each group. The mean age was approximately 63.5 years, roughly 61% were female, and approximately 57% self-identified as White. Before matching, notable imbalances were observed in several covariates, including diabetes mellitus, albumin levels, C-reactive protein, and metformin use. Following matching, all standardized mean differences fell below 0.10, indicating adequate covariate balance across demographic, comorbidity, laboratory, and medication variables (Table 1; Supplementary Table 2). The propensity score density distributions confirmed satisfactory overlap between the two cohorts (Figure 1).

**Table 1:** Baseline characteristics of patients with low vitamin B12 status and matched controls before and after propensity score matching.

Variables	Before matching			After matching		
	Low vitamin B12 group (*n* = 129,162)	Control group (*n* = 612,405)	SMD	Low vitamin B12 group (*n* = 129,159)	Control group (*n* = 129,159)	SMD
Patient characteristics						
Age at index (years)	63.5 ± 12.8	62.4 ± 12.4	0.088	63.5 ± 12.8	63.6 ± 12.6	0.012
BMI ≥ 30 (kg/m^2^)	36,290 (28.1)	161,454 (26.4)	0.039	36,287 (28.1)	35,470 (27.5)	0.014
Female	79,275 (61.4)	387,785 (63.3)	0.040	79,274 (61.4)	78,591 (60.8)	0.011
White	73,204 (56.7)	393,078 (64.2)	0.154	73,204 (56.7)	71,826 (55.6)	0.022
Black or African American	10,965 (8.5)	55,203 (9.0)	0.019	10,965 (8.5)	10,833 (8.4)	0.004
Asian	3,183 (2.5)	17,393 (2.8)	0.023	3,183 (2.5)	3,280 (2.5)	0.005
Comorbidities and healthcare utilization						
Factors influencing health status and contact with health services	68,655 (53.2)	321,542 (52.5)	0.013	68,652 (53.2)	67,307 (52.1)	0.021
Essential (primary) hypertension	49,623 (38.4)	220,243 (36.0)	0.051	49,620 (38.4)	48,224 (37.3)	0.022
Neoplasms	32,879 (25.5)	149,587 (24.4)	0.024	32,876 (25.5)	32,607 (25.2)	0.005
Diabetes mellitus	26,291 (20.4)	98,765 (16.1)	0.110	26,288 (20.4)	25,080 (19.4)	0.023
Vitamin D deficiency	20,853 (16.1)	101,465 (16.6)	0.011	20,853 (16.1)	20,339 (15.7)	0.011
Overweight and obesity	19,973 (15.5)	82,949 (13.5)	0.055	19,971 (15.5)	19,218 (14.9)	0.016
Anxiety, dissociative, stress-related, somatoform and other nonpsychotic mental disorders	19,916 (15.4)	91,463 (14.9)	0.013	19,915 (15.4)	19,028 (14.7)	0.019
Disorders of thyroid gland	19,853 (15.4)	97,583 (15.9)	0.016	19,853 (15.4)	19,356 (15.0)	0.011
Sleep disorders	17,480 (13.5)	82,028 (13.4)	0.004	17,480 (13.5)	16,928 (13.1)	0.013
Mood [affective] disorders	17,304 (13.4)	74,906 (12.2)	0.035	17,302 (13.4)	16,656 (12.9)	0.015
Other anemias	17,032 (13.2)	72,032 (11.8)	0.043	17,030 (13.2)	16,456 (12.7)	0.013
Ischemic heart diseases	14,541 (11.3)	58,006 (9.5)	0.059	14,539 (11.3)	14,134 (10.9)	0.010
Nicotine dependence	11,171 (8.6)	39,936 (6.5)	0.080	11,168 (8.6)	10,710 (8.3)	0.013
Iron deficiency anemia	10,088 (7.8)	38,594 (6.3)	0.059	10,087 (7.8)	9,703 (7.5)	0.011
Noninfective enteritis and colitis	9,638 (7.5)	37,142 (6.1)	0.056	9,637 (7.5)	9,369 (7.3)	0.008
Diseases of liver	8,084 (6.3)	35,836 (5.9)	0.017	8,083 (6.3)	7,804 (6.0)	0.009
COPD	7,429 (5.8)	27,898 (4.6)	0.054	7,427 (5.8)	7,100 (5.5)	0.011
Chronic kidney disease	6,726 (5.2)	26,933 (4.4)	0.038	6,725 (5.2)	6,430 (5.0)	0.010
Atrial fibrillation and flutter	6,440 (5.0)	28,374 (4.6)	0.016	6,440 (5.0)	6,128 (4.7)	0.011
Heart failure	5,479 (4.2)	21,321 (3.5)	0.039	5,478 (4.2)	5,278 (4.1)	0.008
Cerebrovascular diseases	3,528 (2.7)	14,990 (2.4)	0.018	3,528 (2.7)	3,452 (2.7)	0.004
Alcohol related disorders	3,347 (2.6)	14,907 (2.4)	0.010	3,346 (2.6)	3,194 (2.5)	0.007
Systemic connective tissue disorders	2,803 (2.2)	13,026 (2.1)	0.003	2,803 (2.2)	2,696 (2.1)	0.006
COVID-19	2,692 (2.1)	10,599 (1.7)	0.026	2,692 (2.1)	2,598 (2.0)	0.005
Malnutrition	1,521 (1.2)	5,752 (0.9)	0.023	1,521 (1.2)	1,456 (1.1)	0.005

Data are presented as *n* (%) or mean ± SD. An absolute SMD <0.10 was considered adequate balance. BMI, body mass index; COPD, chronic obstructive pulmonary disease; SMD, standardized mean difference.

### 3.2 Primary and secondary outcomes

**Figure 2:**
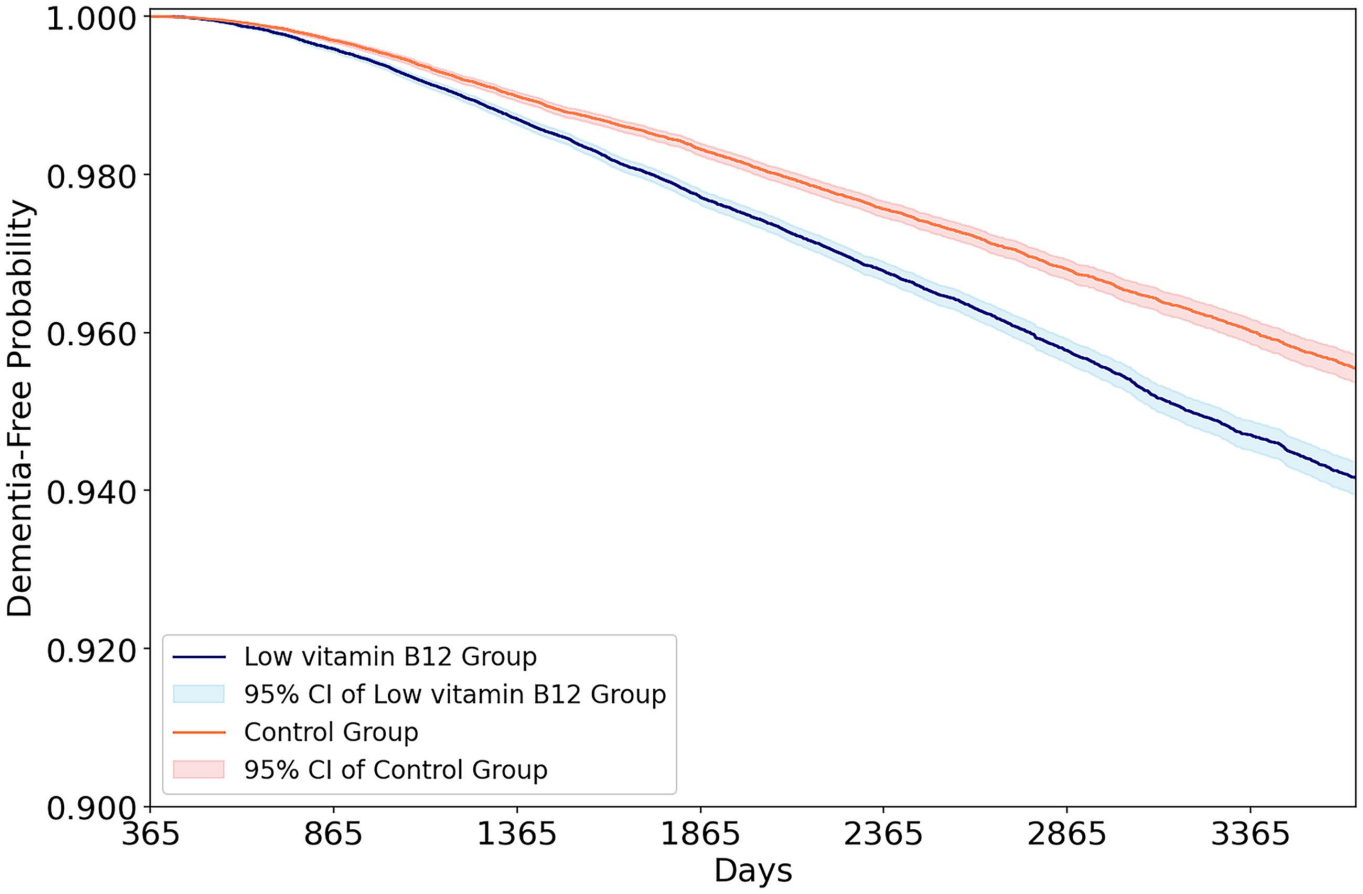
Kaplan–Meier curves for dementia-free survival after propensity score matching. Follow-up began after the 1-year landmark period and continued for up to 10 years. The persistent low vitamin B12 cohort showed lower dementia-free survival than the reference cohort and was associated with a higher risk of incident dementia (HR 1.33, 95% CI 1.27–1.39; *p* < 0.001). The Kaplan–Meier graph data exported from TriNetX included survival probabilities and 95% confidence intervals over time, but did not provide numbers at risk at each time point.

The mean follow-up duration was 5.9 years in the low vitamin B12 cohort and 6.1 years in the reference cohort. Over the 10-year observation period after the 1-year landmark, the low vitamin B12 cohort had a higher incidence of all-cause dementia compared with the reference cohort (2.93% vs. 2.31%; HR 1.33, 95% CI 1.27–1.39; *p* < 0.001) (Table 2). The E-value for the point estimate was 1.99, and the E-value for the lower bound of the confidence interval was 1.86. Although E-values do not exclude residual confounding, these values indicate that an unmeasured confounder would need to be associated with both low vitamin B12 status and incident dementia by a risk ratio of nearly 2.0, beyond measured covariates, to explain away the primary association. Kaplan–Meier analysis showed that patients with low vitamin B12 status had significantly lower dementia-free survival compared with matched controls throughout the observation period (log-rank *p* < 0.001; Figure 2). Among the secondary outcomes, low vitamin B12 status was associated with Alzheimer’s disease (HR 1.34; *p* < 0.001), vascular dementia (HR 1.31; *p* < 0.001), other dementia subtypes (HR 1.32; *p* < 0.001), mild cognitive impairment (HR 1.33; *p* < 0.001), stroke (HR 1.31; *p* < 0.001), and all-cause mortality (HR 1.23; *p* < 0.001) (Table 2).

**Table 2:** Association between low vitamin B12 status and 10-year clinical outcomes.

Outcome	Low vitamin B12 group (*n* = 129,159)	Control group (*n* = 129,159)	HR (95% CI)	*p* value
	Events (%)	Events (%)		
Primary outcome				
All-cause dementia	3,783 (2.93)	2,987 (2.31)	1.33 (1.27–1.39)	<0.001
Secondary outcomes				
Vascular dementia	584 (0.45)	466 (0.36)	1.31 (1.16–1.48)	<0.001
Other type of dementia	3,306 (2.56)	2,629 (2.04)	1.32 (1.25–1.39)	<0.001
Alzheimer’s disease	1,225 (0.95)	957 (0.74)	1.34 (1.23–1.46)	<0.001
Cognitive impairment	1,602 (1.24)	1,269 (0.98)	1.33 (1.23–1.43)	<0.001
Stroke	5,656 (4.38)	4,509 (3.49)	1.31 (1.26–1.37)	<0.001
Mortality	10,708 (8.29)	9,125 (7.07)	1.23 (1.19–1.26)	<0.001
Positive control outcome				
Vitamin B12 deficiency anemia	10,895 (8.44)	6,050 (4.68)	1.89 (1.83–1.95)	<0.001
Negative control outcome				
Appendicitis	463 (0.36)	522 (0.40)	0.92 (0.81–1.04)	0.193

CI, confidence interval; HR, hazard ratio.

### 3.3 Validation of study design

The positive control analysis demonstrated that patients with low vitamin B12 status had a higher rate of subsequent vitamin B12 deficiency anemia (HR 1.89; *p* < 0.001), consistent with the expected clinical sequelae of sustained low vitamin B12 levels (Table 2). The negative control outcome, acute appendicitis, showed no significant between-group difference (HR 0.92; *p* = 0.193), supporting the specificity of the observed associations.

### 3.4 Sensitivity analyses and subgroup analyses

The association between low vitamin B12 status and incident dementia remained consistent across all six sensitivity analyses (Table 3). Point estimates for all-cause dementia ranged from HR 1.26 (Model VI, excluding patients with a history of neoplasm) to HR 1.35 (Model IV, excluding patients with prior albumin < 3.5 g/dL), and all reached statistical significance (*p* < 0.001). The direction and significance of the associations were similarly preserved for dementia subtypes, stroke, and mortality across models (Table 3).

**Table 3:** Sensitivity analyses for the association between low vitamin B12 status and clinical outcomes.

Outcome	Model I HR (95% CI)	Model II HR (95% CI)	Model III HR (95% CI)	Model IV HR (95% CI)	Model V HR (95% CI)	Model VI HR (95% CI)
All-cause dementia	1.29 (1.22–1.37)	1.32 (1.26–1.39)	1.27 (1.20–1.33)	1.35 (1.28–1.43)	1.31 (1.24–1.38)	1.26 (1.19–1.34)
Vascular dementia	1.32 (1.14–1.53)	1.31 (1.16–1.48)	1.45 (1.27–1.66)	1.36 (1.17–1.57)	1.37 (1.20–1.57)	1.43 (1.22–1.67)
Other type of dementia	1.29 (1.21–1.38)	1.32 (1.26–1.39)	1.25 (1.18–1.32)	1.34 (1.26–1.42)	1.32 (1.25–1.39)	1.26 (1.18–1.35)
Alzheimer’s disease	1.29 (1.17–1.42)	1.37 (1.26–1.49)	1.27 (1.16–1.39)	1.31 (1.19–1.44)	1.29 (1.18–1.41)	1.22 (1.10–1.35)
Cognitive impairment	1.27 (1.18–1.38)	1.28 (1.19–1.38)	1.23 (1.13–1.33)	1.40 (1.29–1.53)	1.27 (1.17–1.37)	1.31 (1.18–1.46)
Stroke	1.31 (1.25–1.37)	1.29 (1.24–1.34)	1.32 (1.27–1.38)	1.36 (1.30–1.43)	1.31 (1.25–1.36)	1.30 (1.23–1.37)
Mortality	—	1.23 (1.19–1.26)	1.22 (1.18–1.25)	1.30 (1.25–1.35)	1.21 (1.17–1.25)	1.17 (1.12–1.21)

Model I excluded patients who died during follow-up; Model II was restricted to patients with >1 healthcare visit during follow-up; Model III excluded patients who developed vitamin B12 deficiency anemia; Model IV excluded patients with prior albumin <3.5 g/dL; Model V excluded patients with prior chronic kidney disease; Model VI excluded patients with prior neoplasm. CI, confidence interval; HR, hazard ratio.

In sex-stratified subgroup analyses, low vitamin B12 status was associated with a higher risk of all-cause dementia in both males (HR 1.33; *p* < 0.001) and females (HR 1.31; *p* < 0.001), with no statistically significant interaction between sex and vitamin B12 status (*p* for interaction = 0.760) (Table 4). The pattern of associations for dementia subtypes, stroke, and mortality was broadly consistent across sexes, and no significant interactions were detected for any outcome.

**Table 4:** Sex-stratified subgroup analyses of low vitamin B12 status and clinical outcomes.

Outcomes	Male (*n* = 50,052 for each group)		Female (*n* = 79,562 for each group)		*p* for interaction
	HR (95% CI)	*p* value	HR (95% CI)	*p* value	
All-cause dementia	1.33 (1.23–1.43)	<0.001	1.31 (1.23–1.39)	<0.001	0.760
Vascular dementia	1.55 (1.28–1.88)	<0.001	1.26 (1.08–1.48)	0.004	0.115
Other type of dementia	1.30 (1.20–1.41)	<0.001	1.31 (1.23–1.40)	<0.001	0.885
Alzheimer’s disease	1.27 (1.11–1.46)	0.001	1.34 (1.20–1.49)	<0.001	0.546
Cognitive impairment	1.39 (1.24–1.57)	<0.001	1.25 (1.13–1.37)	<0.001	0.179
Stroke	1.34 (1.26–1.42)	<0.001	1.32 (1.26–1.39)	<0.001	0.704
Mortality	1.21 (1.16–1.26)	<0.001	1.25 (1.20–1.30)	<0.001	0.268

HR, hazard ratio; CI, confidence interval.

### 3.5 Adjusted hazard ratios from multivariable cox regression

Sequential multivariable Cox regression analyses supported the robustness of the association between low vitamin B12 status and incident all-cause dementia (Table 5). After adjustment for age, sex, and race/ethnicity in the minimally adjusted model, low vitamin B12 status remained associated with a higher risk of dementia, with an adjusted HR of 1.36 (*p* < 0.001). The effect estimate was only modestly attenuated after additional adjustment for cardiometabolic risk factors, systemic comorbidities, neuropsychiatric conditions, and chronic diseases. In the fully adjusted model, the association remained statistically significant, with an adjusted HR of 1.32 (*p* < 0.001).

**Table 5:** Sequential multivariable Cox models for incident dementia.

Model	Adjusted HR (95% CI)	*p*-value	Covariates included in the model
Model 1	1.36 (1.31–1.41)	<0.001	Sex, age at index, race
Model 2	1.35 (1.30–1.40)	<0.001	Model 1 + hypertension, ischemic heart disease, atrial fibrillation/flutter, heart failure, cerebrovascular disease
Model 3	1.33 (1.28–1.37)	<0.001	Model 2 + diabetes mellitus, overweight/obesity, nicotine dependence, alcohol-related disorders, thyroid disorders
Model 4	1.32 (1.28–1.37)	<0.001	Model 3 + anxiety/stress-related disorders, sleep disorders, mood disorders
Model 5	1.32 (1.28–1.37)	<0.001	Model 4 + neoplasms, liver disease, chronic kidney disease

Models were sequentially adjusted for demographics, cardiovascular diseases, metabolic risk factors, neuropsychiatric conditions, and systemic comorbidities. CI, confidence interval; HR, hazard ratio.

### 3.6 Exposure-gradient analysis: association between vitamin B12 deficiency and dementia risk

Among patients with vitamin B12 deficiency (< 200 pg./mL), 23,065 patients were matched 1:1 with 23,065 reference patients. The vitamin B12 deficiency cohort had a higher incidence of all-cause dementia compared with the reference cohort (3.24% vs. 2.18%; HR 1.64, 95% CI 1.46–1.84; *p* < 0.001) (Table 6). This magnitude of association was numerically larger than that observed in the primary analysis (HR 1.33), suggesting a potential exposure-gradient relationship. The associations with all secondary outcomes were also stronger in the deficiency cohort, with HRs of 1.61 for vascular dementia, 1.52 for Alzheimer’s disease, 1.62 for other dementia subtypes, 1.36 for mild cognitive impairment, 1.49 for stroke, and 1.43 for all-cause mortality (all *p* < 0.001). The positive control outcome, vitamin B12 deficiency anemia, showed a substantially stronger association (HR 2.70; *p* < 0.001) than in the primary analysis (HR 1.89), while the negative control outcome remained nonsignificant (HR 0.88; *p* = 0.440) (Table 6).

**Table 6:** Exposure-gradient analysis of vitamin B12 deficiency and 10-year clinical outcomes.

Outcome	Vitamin B12 deficiency group (*n* = 23,065)	Control group (*n* = 23,065)	HR (95% CI)	*p* value
	Events (%)	Events (%)		
Primary outcome				
All-cause dementia	747 (3.24)	503 (2.18)	1.64 (1.46–1.84)	<0.001
Secondary outcomes				
Vascular dementia	116 (0.50)	80 (0.35)	1.61 (1.21–2.13)	0.001
Other type of dementia	651 (2.82)	444 (1.93)	1.62 (1.44–1.83)	<0.001
Alzheimer’s disease	225 (0.98)	163 (0.71)	1.52 (1.25–1.86)	<0.001
Cognitive impairment	312 (1.35)	254 (1.10)	1.36 (1.15–1.61)	<0.001
Stroke	1,105 (4.79)	808 (3.50)	1.49 (1.36–1.64)	<0.001
Mortality	2,221 (9.63)	1,699 (7.37)	1.43 (1.34–1.52)	<0.001
Positive control outcome				
Vitamin B12 deficiency anemia	2,684 (11.64)	1,088 (4.72)	2.70 (2.52–2.90)	<0.001
Negative control outcome				
Appendicitis	67 (0.29)	82 (0.36)	0.88 (0.64–1.22)	0.440

CI, confidence interval; HR, hazard ratio. Vitamin B12 deficiency was defined as two vitamin B12 measurements <200 pg/mL.

## 4 Discussion

Whether low vitamin B12 status is independently associated with long-term dementia risk has remained uncertain, largely because of methodological heterogeneity across prior studies. In this large-scale propensity score–matched cohort study, low vitamin B12 status, was associated with a higher 10-year risk of incident all-cause dementia after a 1-year landmark period. The association was observed across dementia subtypes, mild cognitive impairment, stroke, and all-cause mortality, with evidence of an exposure-gradient relationship among patients with more severe deficiency.

The present findings contrast with those of Arendt et al. (18) who reported no association between low plasma vitamin B12 levels and dementia risk in a Danish registry-based cohort of over 53,000 propensity score–matched patients followed for up to 15 years. Several methodological differences may help explain this discrepancy. First, Arendt et al. (18) classified exposure based on a single vitamin B12 measurement, which may not distinguish persistent deficiency from transient laboratory variation. The present study required two qualifying measurements within a 2-year window, thereby increasing the likelihood that the exposure reflects a sustained state. Second, the absence of a lag or landmark period in the Danish study (18) may have increased susceptibility to reverse causation, because early dementia diagnoses could reflect pre-existing or prodromal neurocognitive decline rather than dementia developing after vitamin B12 assessment. Such events may have biased the estimated association toward the null or contributed to inconsistent findings.

Nevertheless, our exposure definition may have selected patients with greater healthcare contact, thereby introducing potential ascertainment bias. In addition, requiring two vitamin B12 measurements within two years may have introduced selection bias by preferentially including patients who were more closely monitored, more engaged with healthcare services, or perceived by clinicians as having nutritional, hematologic, neurologic, or systemic conditions requiring follow-up testing. Such patients may differ systematically from individuals with only one vitamin B12 measurement, and this design feature may limit the generalizability of the findings to all patients with low vitamin B12 levels. To partially address this concern, we performed a sensitivity analysis restricted to patients with more than one healthcare visit during follow-up (Model II), in which the association between low vitamin B12 status and incident dementia remained materially unchanged. Although this analysis cannot fully eliminate selection bias related to repeat testing, it suggests that the observed association was not explained solely by differential healthcare contact during follow-up. Nevertheless, residual surveillance bias remains possible because closer clinical monitoring may have increased the likelihood of dementia or cognitive impairment detection in the low vitamin B12 cohort. Importantly, this repeated-testing strategy likely defines a clinically enriched and medically engaged population rather than a general community-based population. Therefore, the findings should be generalized primarily to patients undergoing repeated vitamin B12 assessment in routine healthcare settings, rather than to all older adults with possible low vitamin B12 status.

Although the 1-year landmark period improved temporal separation between vitamin B12 assessment and dementia ascertainment, reverse causation cannot be fully excluded in current study. Dementia has a prolonged preclinical phase, during which subtle cognitive decline, frailty, weight loss, reduced dietary intake, and poorer treatment adherence may precede formal diagnosis and contribute to low vitamin B12 status. Therefore, low vitamin B12 may partly represent an early marker of neurodegenerative or nutritional decline rather than an upstream causal exposure. Longer lag periods may further reduce this bias, but were not performed because of concerns about reduced event numbers and follow-up duration. In addition, serum vitamin B12 concentration alone may not fully capture biologically meaningful vitamin B12 deficiency. Functional deficiency can occur despite serum values within the conventional reference range, whereas low serum levels may not necessarily indicate tissue-level deficiency. Because methylmalonic acid, homocysteine, and holotranscobalamin were unavailable in TriNetX, exposure misclassification remains possible. Future studies incorporating functional biomarkers are needed to better define clinically relevant vitamin B12 deficiency and its association with dementia risk.

The importance of repeated measurements for characterizing vitamin B12 status is supported by recent findings from the Framingham Heart Study, which used at least two assessments of a composite vitamin B12 indicator and reported that higher cumulative vitamin B12 status from midlife to late life was associated with slower cognitive decline (27). Although that study used a composite biomarker rather than serum vitamin B12 concentrations, its findings are broadly consistent with the present association between sustained low vitamin B12 status and adverse neurocognitive outcomes. Meta-analyses examining the relationship between serum vitamin B12 and dementia risk have yielded inconsistent results, a discrepancy attributed in part to heterogeneity in exposure definitions, follow-up durations, and the absence of lag periods (16, 17).

A notable observation was the broadly consistent association across dementia subtypes. The HRs for Alzheimer’s disease, vascular dementia, and other dementia categories were similar in magnitude, ranging from 1.31 to 1.34. This pattern may suggest that persistent low vitamin B12 status is not limited to a single dementia subtype, although subtype-specific findings should be interpreted cautiously because dementia classification based on diagnostic codes may be imperfect. The relatively uniform effect sizes across subtypes may be compatible with biological pathways shared across neurodegenerative and vascular processes, including impaired one-carbon metabolism, homocysteine-mediated neurotoxicity, endothelial dysfunction, and disrupted myelin maintenance (7, 11–14). The comparable association observed for stroke as an independent secondary outcome is consistent with, but does not establish, a possible vascular component of the observed association. However, the present study was not designed to determine whether cerebrovascular events mediate the relationship between low vitamin B12 status and dementia risk.

The exposure-gradient analysis provided additional support for the observed associations. Among patients with vitamin B12 deficiency (<200 pg./mL), the HR for all-cause dementia was 1.64, compared with 1.33 in the primary analysis. A parallel gradient was observed for the positive control outcome, with the HR for vitamin B12 deficiency anemia increasing from 1.89 to 2.70. In contrast, the negative control outcome remained nonsignificant at both exposure thresholds, which reduces the likelihood that the findings were driven solely by nonspecific ascertainment bias or generalized residual confounding. However, this exposure-gradient finding should be interpreted cautiously because it was based on a smaller subgroup, the confidence intervals overlapped with those of the primary analysis, and no formal trend test was performed. This numerically stronger association among patients with vitamin B12 deficiency should be interpreted as exploratory and hypothesis-generating, rather than as evidence of a dose–response or causal relationship. Nevertheless, the primary association represented a modest absolute risk difference, with 10-year dementia incidences of 2.93 and 2.31% in the low vitamin B12 and reference cohorts, respectively. Therefore, the findings should be interpreted as a modest epidemiologic signal rather than evidence of a large causal effect or a major modifiable determinant of dementia risk.

Several sensitivity analyses supported the robustness of the primary findings. The association persisted after excluding patients who died during follow-up, those with chronic kidney disease, and those with a history of neoplasm. After excluding patients with prior albumin levels below 3.5 g/dL, the HR for dementia was numerically the highest among all models (HR 1.35). Although this difference may partly reflect changes in sample composition, the persistence of the association in a subgroup less likely to have overt hypoalbuminemia provides some reassurance that the findings were not driven solely by general malnutrition or severe systemic illness. Furthermore, the association remained significant after excluding patients who developed vitamin B12 deficiency anemia during follow-up (HR 1.27), suggesting that the observed relationship was not limited to patients with clinically apparent hematologic manifestations of vitamin B12 deficiency. In sex-stratified analyses, low vitamin B12 status was associated with dementia in both males and females, with no significant interaction by sex. The numerically stronger association with vascular dementia in males (HR 1.55 vs. 1.26 in females) should be interpreted cautiously but may warrant further investigation.

This study has several limitations. First, because of its observational design, residual confounding cannot be excluded. Functional vitamin B12 biomarkers, including homocysteine and methylmalonic acid, were unavailable, and data on dietary patterns, socioeconomic status, apolipoprotein E genotype, over-the-counter supplementation, and vitamin B12 treatment during follow-up were not captured. These unmeasured factors may have either inflated the observed association, if low vitamin B12 status was correlated with poorer health or frailty, or attenuated it, if patients with low vitamin B12 received supplementation after baseline assessment. Because information on oral or injection vitamin B12 replacement, treatment adherence, and post-index correction of vitamin B12 status was unavailable, time-updated exposure modeling could not be performed, and exposure misclassification during follow-up remains possible. Second, although TriNetX includes a large international network, participating healthcare organizations are predominantly located in high-income healthcare settings, and the matched cohort was predominantly White and medically engaged. Thus, generalizability may be limited to lower-resource settings, nutritionally vulnerable populations, or community-based adults with different dietary patterns, healthcare access, or vitamin B12 deficiency prevalence. Third, dementia ascertainment relied on ICD-10-CM diagnostic codes, which may underestimate incidence and imperfectly distinguish Alzheimer’s disease, vascular dementia, and other dementia subtypes. In addition, delayed diagnosis, under-recognition of mild or early dementia, variable coding practices across healthcare organizations, and limited specificity of subtype coding may have introduced outcome misclassification. These limitations are particularly relevant to vascular dementia and mild cognitive impairment, for which coding accuracy may be less consistent in routine clinical practice. Fourth, the cause-specific Cox model estimates etiological associations but does not directly estimate cumulative incidence in the presence of competing mortality. Because mortality exceeded dementia incidence, death may have affected absolute risk interpretation. Fine–Gray modeling could provide complementary competing-risk estimates; however, this analysis was not performed because the TriNetX analytics platform does not provide Fine–Gray subdistribution hazard modeling within its built-in propensity score–matched survival analysis module. Therefore, our findings should be interpreted as etiologic cause-specific associations rather than competing-risk-adjusted absolute risk estimates. Fifth, although the primary analysis focused on incident all-cause dementia, multiple secondary, sensitivity, subgroup, control, and exposure-gradient analyses were also performed without multiplicity adjustment. Therefore, statistically significant findings from these non-primary analyses should be interpreted as exploratory and hypothesis-generating rather than confirmatory. In addition, because the large sample size may yield statistically significant results despite modest absolute differences, clinical relevance should be judged by the magnitude and consistency of effect estimates, absolute risks, and biological plausibility rather than *p* values alone. Finally, co-existing micronutrient abnormalities, such as folate, vitamin D, magnesium, and iron status, were not fully captured and may contribute to mutual confounding through shared links with malnutrition, inflammation, frailty, and healthcare utilization.

## 5 Conclusion

This propensity score–matched study observed an association between persistent low vitamin B12 status and increased long-term risk of incident dementia, with a numerically stronger association among patients with vitamin B12 deficiency. Given the observational design and the potential for residual confounding, reverse causation, and outcome ascertainment bias, this association should be interpreted as a modest epidemiologic signal rather than evidence of causality. Further prospective studies with serial biomarker monitoring and randomized trials are needed to determine whether correction of low vitamin B12 status can influence dementia risk.

## Data Availability

The data used in this study were obtained from the TriNetX Global Collaborative Network under license and are not publicly available. Patient-level data cannot be exported from the TriNetX federated platform or shared by the authors. Aggregate-level reproducibility materials, including cohort definitions, code lists, outcome definitions, time-window specifications, propensity score matching covariates, and aggregate outcome estimates, are available from the corresponding author upon reasonable request.
