# Supplementary material for: Association of low vitamin B12 status with incident dementia and stroke: an EHR database study
Source: Front Nutr. 2026 Jul 7;13:1877529. doi: 10.3389/fnut.2026.1877529 (PMC13385101; doi:10.3389/fnut.2026.1877529)
Supplement: Supplementary file 1 [file Table_1.DOCX]

**Supplemental Table 1. Codes Used for Cohort Definition, Inclusion/Exclusion Criteria, Outcome Definitions, and Variables for Propensity Score Matching**

| Domain | Variable / Definition | Code / Term |
| --- | --- | --- |
| Cohort definition | Age | ≥50 years |
|  | Persistent low vitamin B12 status | TNX:9065; cobalamin/vitamin B12 in serum, plasma, or blood ≤299 pg/mL; two measurements within 2 years |
|  | Reference vitamin B12 status | TNX:9065; cobalamin/vitamin B12 in serum, plasma, or blood 300–900 pg/mL; two measurements within 2 years |
| Acute illness exclusion near vitamin B12 measurement | Acute kidney failure | ICD-10-CM N17 |
|  | Sepsis | ICD-10-CM A41 |
|  | Severe sepsis | ICD-10-CM R65.2 |
|  | Critical care services | CPT 1013729 |
| Baseline exclusion criteria | Prior dementia | ICD-10-CM F01, F02, F03, G30–G32 |
|  | Advanced chronic kidney disease / end-stage renal disease / dialysis dependence | ICD-10-CM N18.4, N18.5, N18.6, Z99.2 |
|  | Parkinson’s disease | ICD-10-CM G20 |
|  | Bipolar disorder | ICD-10-CM F31 |
|  | Schizophrenia, schizotypal, delusional, and other non-mood psychotic disorders | ICD-10-CM F20–F29 |
|  | Prior cerebrovascular disease | ICD-10-CM I61, I63, G45 |
|  | Bariatric surgery status / bariatric surgery procedures | ICD-10-CM Z98.84; CPT 1007385 |
|  | Malignant neoplasm of meninges | ICD-10-CM C70 |
|  | Malignant neoplasm of brain | ICD-10-CM C71 |
|  | Malignant neoplasm of spinal cord, cranial nerves, and other CNS sites | ICD-10-CM C72 |
|  | Benign neoplasm of meninges | ICD-10-CM D32 |
|  | Benign neoplasm of brain and other CNS sites | ICD-10-CM D33 |
|  | Neoplasm of uncertain behavior of meninges | ICD-10-CM D42 |
|  | Neoplasm of unspecified behavior of brain | ICD-10-CM D49.6 |
|  | Intracranial injury | ICD-10-CM S06 |
| Primary outcome | Overall dementia | ICD-10-CM F01, F02, F03, G30 |
| Secondary outcomes | Alzheimer’s disease | ICD-10-CM G30 |
|  | Vascular dementia | ICD-10-CM F01 |
|  | Other/unspecified dementia | ICD-10-CM F02, F03 |
|  | Mild cognitive impairment | ICD-10-CM G31.84 |
|  | Stroke | ICD-10-CM I63, G45 |
|  | All-cause mortality | Deceased status; ICD-10-CM R99 |
| Control outcomes | Vitamin B12 deficiency anemia | ICD-10-CM D51 |
|  | Acute appendicitis | ICD-10-CM K35 |
| Comorbidities for propensity score matching | Hypertension | ICD-10-CM I10 |
|  | Overweight and obesity | ICD-10-CM E66 |
|  | Diabetes mellitus | ICD-10-CM E08–E13 |
|  | Nicotine dependence | ICD-10-CM F17 |
|  | Ischemic heart disease | ICD-10-CM I20–I25 |
|  | Chronic kidney disease | ICD-10-CM N18 |
|  | Alcohol-related disorders | ICD-10-CM F10 |
|  | Cerebrovascular diseases | ICD-10-CM I60–I69 |
|  | Chronic obstructive pulmonary disease | ICD-10-CM J44 |
|  | Malnutrition | ICD-10-CM E40–E46 |
|  | Factors influencing health status and contact with health services | ICD-10-CM Z00–Z99 |
|  | Sleep disorders | ICD-10-CM G47 |
|  | Heart failure | ICD-10-CM I50 |
|  | Atrial fibrillation and flutter | ICD-10-CM I48 |
|  | Systemic connective tissue disorders | ICD-10-CM M30–M36 |
|  | Mood disorders | ICD-10-CM F30–F39 |
|  | Anxiety, stress-related, somatoform, and other nonpsychotic mental disorders | ICD-10-CM F40–F48 |
|  | Liver diseases | ICD-10-CM K70–K77 |
|  | Thyroid disorders | ICD-10-CM E00–E07 |
|  | Vitamin D deficiency | ICD-10-CM E55 |
|  | Neoplasms | ICD-10-CM C00–D49 |
|  | COVID-19 | ICD-10-CM U07.1 |
|  | Other anemias | ICD-10-CM D64 |
|  | Iron deficiency anemia | ICD-10-CM D50 |
|  | Noninfective enteritis and colitis | ICD-10-CM K50–K52 |
| Medications for propensity score matching | Benzodiazepine derivative sedatives/hypnotics | RxNorm / ATC class CN302 |
|  | Insulins and analogues | ATC A10A |
|  | Central nervous system medications | RxNorm / ATC class CN000 |
|  | Diuretics | RxNorm / ATC class CV700 |
|  | Anticholinergics | ATC S01FA |
|  | Anticonvulsants | RxNorm / ATC class CN400 |
|  | Proton pump inhibitors | ATC A02BC |
|  | Metformin | RxNorm 6809 |
|  | Sodium-glucose cotransporter 2 inhibitors | ATC A10BK |
| Laboratory/body-composition variables for propensity score matching | Albumin | TNX 9045 |
|  | Albumin ≥3.5 g/dL | TNX 9045; value ≥3.5 g/dL |
|  | Estimated glomerular filtration rate | LOINC 98979-8 |
|  | eGFR ≥60 mL/min/1.73 m² | LOINC 98979-8; value ≥60 mL/min/1.73 m² |
|  | Hemoglobin A1c | TNX 9037 |
|  | Hemoglobin A1c ≥9% | TNX 9037; value ≥9% |
|  | C-reactive protein | TNX 9063 |
|  | C-reactive protein ≥10 mg/L | TNX 9063; value ≥10 mg/L |
|  | Hemoglobin | TNX 9014 |
|  | Hemoglobin ≥12 g/dL | TNX 9014; value ≥12 g/dL |
|  | Body mass index | TNX 9083 |
|  | BMI ≥30 kg/m² | TNX 9083; value ≥30 kg/m² |
|  | Magnesium | TNX 9026 |
|  | Magnesium 1.70–2.20 mg/dL | TNX 9026; value 1.70–2.20 mg/dL |

**Supplemental table 2.** **Baseline laboratory data and medication exposures before and after propensity score matching**

| Variables | Before matching | | | After matching | | | |
| --- | --- | --- | --- | --- | --- | --- | --- |
|  | Low vitamin B12 group  (n = 129,162) | Control group  (n = 612,405) | SMD | | Low vitamin B12 group  (n = 129,159) | Control group  (n = 129,159) | SMD |
| Laboratory data |  |  |  | |  |  |  |
| Hemoglobin ≥ 12 g/dL | 85158 (65.9) | 388699 (63.5) | 0.051 | | 85155 (65.9) | 85428 (66.1) | 0.004 |
| Albumin ≥ 3.5 g/dL | 80937 (62.7) | 352972 (57.6) | 0.103 | | 80934 (62.7) | 80761 (62.5) | 0.003 |
| HbA1c ≥ 9% | 5732 (4.4) | 20658 (3.4) | 0.055 | | 5730 (4.4) | 5552 (4.3) | 0.007 |
| eGFR ≥60 mL/min/1.73 m² | 82338 (63.7) | 392872 (64.2) | 0.008 | | 82335 (63.7) | 82689 (64.0) | 0.006 |
| C-reactive protein ≥ 10 mg/L | 13533 (10.5) | 44961 (7.3) | 0.110 | | 13530 (10.5) | 13418 (10.4) | 0.003 |
| Magnesium 1.7-2.2 mg/dL | 21760 (16.8) | 91628 (15.0) | 0.052 | | 21757 (16.8) | 21026 (16.3) | 0.015 |
| Medications |  |  |  | |  |  |  |
| Central nervous system medications | 67701 (52.4) | 309784 (50.6) | 0.037 | | 67698 (52.4) | 66629 (51.6) | 0.017 |
| Proton pump inhibitors | 32047 (24.8) | 139399 (22.8) | 0.048 | | 32045 (24.8) | 31183 (24.1) | 0.016 |
| Benzodiazepine | 31973 (24.8) | 145055 (23.7) | 0.025 | | 31970 (24.8) | 31196 (24.2) | 0.014 |
| Diuretics | 25756 (19.9) | 111758 (18.2) | 0.043 | | 25754 (19.9) | 25043 (19.4) | 0.014 |
| Anticonvulsants | 16632 (12.9) | 67527 (11.0) | 0.057 | | 16629 (12.9) | 15987 (12.4) | 0.015 |
| Metformin | 16016 (12.4) | 54967 (9.0) | 0.111 | | 16013 (12.4) | 15081 (11.7) | 0.022 |
| Insulins and analogues | 11712 (9.1) | 44971 (7.3) | 0.063 | | 11709 (9.1) | 11235 (8.7) | 0.013 |
| Anticholinergics | 8868 (6.9) | 35524 (5.8) | 0.044 | | 8865 (6.9) | 8664 (6.7) | 0.006 |
| SGLT2 inhibitors | 2427 (1.9) | 8649 (1.4) | 0.037 | | 2426 (1.9) | 2325 (1.8) | 0.006 |

Data are presented as n (%) or mean ± SD. An absolute SMD <0.10 was considered adequate balance. eGFR, estimated glomerular filtration rate; HbA1c, hemoglobin A1c; SMD, standardized mean difference. SGLT2: Sodium-glucose co-transporter 2
